# Genetic Determinism of Sensitivity to *Corynespora cassiicola* Exudates in Rubber Tree (*Hevea brasiliensis*)

**DOI:** 10.1371/journal.pone.0162807

**Published:** 2016-10-13

**Authors:** Dinh Minh Tran, André Clément-Demange, Marine Déon, Dominique Garcia, Vincent Le Guen, Anne Clément-Vidal, Mouman Soumahoro, Aurélien Masson, Philippe Label, Mau Tuy Le, Valérie Pujade-Renaud

**Affiliations:** 1 Rubber Research Institute of Vietnam, Ho Chi Minh City, Vietnam; 2 CIRAD, UMR-AGAP, F-34398 Montpellier, France; 3 UCA, INRA, UMR PIAF, 63000 Clermont-Ferrand, France; 4 Société Africaine de Plantations d'Hévéas, 01 BP 1322 Abidjan 01, Côte d’Ivoire; 5 Société des Caoutchoucs de Grand-Béréby, Grand-Béréby, Côte d’Ivoire; 6 CIRAD, UMR-AGAP, F-63000 Clermont-Ferrand, France; Universita degli Studi di Pisa, ITALY

## Abstract

An indirect phenotyping method was developed in order to estimate the susceptibility of rubber tree clonal varieties to *Corynespora* Leaf Fall (CLF) disease caused by the ascomycete *Corynespora cassiicola*. This method consists in quantifying the impact of fungal exudates on detached leaves by measuring the induced electrolyte leakage (EL%). The tested exudates were either crude culture filtrates from diverse *C*. *cassiicola* isolates or the purified cassiicolin (Cas1), a small secreted effector protein produced by the aggressive isolate CCP. The test was found to be quantitative, with the EL% response proportional to toxin concentration. For eight clones tested with two aggressive isolates, the EL% response to the filtrates positively correlated to the response induced by conidial inoculation. The toxicity test applied to 18 clones using 13 toxinic treatments evidenced an important variability among clones and treatments, with a significant additional clone x treatment interaction effect. A genetic linkage map was built using 306 microsatellite markers, from the F1 population of the PB260 x RRIM600 family. Phenotyping of the population for sensitivity to the purified Cas1 effector and to culture filtrates from seven *C*. *cassiicola* isolates revealed a polygenic determinism, with six QTL detected on five chromosomes and percentages of explained phenotypic variance varying from 11 to 17%. Two common QTL were identified for the CCP filtrate and the purified cassiicolin, suggesting that Cas1 may be the main effector of CCP filtrate toxicity. The CCP filtrate clearly contrasted with all other filtrates. The toxicity test based on Electrolyte Leakage Measurement offers the opportunity to assess the sensitivity of rubber genotypes to *C*. *cassiicola* exudates or purified effectors for genetic investigations and early selection, without risk of spreading the fungus in plantations. However, the power of this test for predicting field susceptibility of rubber clones to CLF will have to be further investigated.

## Introduction

The rubber tree (*Hevea brasiliensis*, 2n = 36) is the only crop developed for the production of natural rubber. With a total of twelve million tons on about 12 million hectares in 2013 (http://faostat3.fao.org/), natural rubber production is mostly concentrated in Asia (92%), followed by West Africa (5%). Latex is collected regularly by incision of the trunk bark, on trees older than 5–6 years and during 20–30 years. *Hevea* spp. are monoecious and allogamous. *H*. *brasiliensis* selected elite genotypes are vegetatively propagated by grafting onto seedling rootstocks. Early selection methods are required to assess the main performances of rubber clones prior to long-lasting agronomical studies of the most promising candidates. Rubber tree is affected by several important cryptogamic diseases. In Latin America, from which *Hevea* originated, rubber cropping is severely hampered by the South American Leaf Blight, caused by *Microcyclus ulei*, alias *Pseudocercospora ulei* [1]. However, this disease remains so far confined to that continent. In Asia and Africa, one of the most important diseases is Corynespora Leaf Fall (CLF), caused by *Corynespora cassiicola* (Berk. & M. T. Curtis), C. T. Wei. This ascomycete fungus was first isolated from rubber tree in Sierra Leone [2], then in India [3] and Malaysia [4]. It was really considered dangerous after the epidemic outbreak in Sri Lanka in 1985 [5]. CLF disease is characterized by the development of necrotic lesions on the leaves with frequent blackening of the veins giving a typical “fish-bone” or “railway track” appearance. Both immature and mature leaves are affected, leading to massive defoliation and consequently growth delay and yield losses. Chemical control is technically feasible in nursery, but not sustainable in plantation and harmful to the workers and the environment. Early detection and exclusion of highly susceptible clones is essential to avoid inoculum proliferation and epidemic outbreaks.

Studies in controlled conditions have highlighted the role of a small secreted phytotoxic protein involved in *C*. *cassiicola* pathogenicity in rubber tree [6–8]. This toxin, named cassiicolin, was purified from a highly aggressive pathogenic isolate, CCP, collected from rubber plantations in the Philippines. It is a glycosylated cysteine-rich small secreted protein (CR-SSP) of 27 amino acids. Rubber clones were tested for their sensitivity to the purified Cas1 using a leaf wilting assay in comparison with conidial inoculation of CCP [6]. The profiles obtained in both cases were in good agreement, suggesting that cassiicolin plays an important role in the virulence of the fungus.

The cassiicolin-encoding gene was found to be transiently expressed in the early phase of infection, just before the occurrence of the first symptoms [9]. Six cassiicolin isoforms (Cas1, Cas2, Cas3, Cas4, Cas5 and Cas6) were identified by PCR-detection on a collection of *C*. *cassiicola* strains from various host plants and geographical origins [10]. In rare cases, two *Cas* genes (encoding Cas2 and Cas6 isoforms respectively) were detected in the same isolate. However, 53% of the tested isolates had no detectable *Cas* gene, although some of them were virulent in rubber tree, suggesting that effectors other than cassiicolin may be involved in their virulence. The isolates were classified into six toxin classes based on their *Cas* gene profile: Cas0 (no detectable *Cas* gene), Cas1, Cas2, Cas3, Cas4, Cas5 and Cas2+6.

Several diversity studies have delineated genetic races within the *C*. *cassiicola* species. However, the concordance of these genetic races with geographical origins or biological traits such as pathogenicity, virulence and host-specificity remains unclear [10–18]. The large genetic diversity of *C*. *cassiicola* is not structured by strict host-specificities but rather host-specialization, with isolates often able to infect a restricted range of plants rather than a single host [15,17]. We have performed a phylogenetic analysis on 129 *C*. *cassiicola* isolates, including 71 isolates from rubber tree, from Asian, African and South American countries [10]. Our study revealed at least eight major phylogenetic clades, in rather good coherence with the toxin classes assigned based on the detection and polymorphism of the *Cas* genes. Indeed, all isolates of the same cassiicolin toxin class were grouped within a single genetic clade; only the Cas0 isolates were scattered in several genetic groups.

The genetic determinism of rubber tree tolerance/susceptibility to *C*. *cassiicola* was so far unknown. Previous studies involving QTL approaches had analyzed the genetic determinism of rubber tree tolerance to the South American Leaf Blight (SALB) [19–23] and were later extended to other traits such as growth speed and latex production (unpublished results). Concerning the *H*. *brasiliensis* x *C*. *cassiicola* interaction, studies were so far limited by the lack of an effective procedure for phenotyping the susceptibility of rubber clones that would take into account the diversity of the pathogen. Recognition and scoring of the disease in natural conditions is difficult considering the diversity of symptoms attributed to *C*. *cassiicola* and the possible confusion with other leaf diseases. The sporadic (epidemic) occurrence of the disease is another difficulty for field observation. Several studies have assessed rubber clones resistance/susceptibility in fields and nurseries [5,24,25], but they were of course limited to the local inoculum, thus making extrapolation unreliable.

Inoculation experiments in controlled conditions are largely limited by the difficulty to combine a large panel of rubber tree clones with a large panel of isolates representative of *C*. *cassiicola* diversity, without taking any risk of contamination; indeed, introducing new virulent isolates for experimentation in rubber-tree growing countries is of course strictly prohibited. An indirect procedure was thus proposed, consisting in testing the sensitivity of rubber clones to fungal exudates rather than susceptibility to the fungus itself [6]. In the initial procedure, the toxicity was either assessed visually (estimation of the leaf necrosis area) or by the leaf wilting assay (measuring water losses). However, the sensitivity of this test for a large range of isolates and clones, and its capacity to predict the sensitivity in field conditions, was questioned [26].

In this article, we present (i) the set-up and validation of an improved phenotyping method based on the response of rubber clones to *C*. *cassiicola* exudates (culture filtrate or purified toxin), by conductivity measurement of the induced electrolyte leakage, and (ii) the genetic mapping and phenotyping of a biparental rubber tree family for the detection of QTL involved in the sensitivity/tolerance to *C*. *cassiicola* exudates.

## Materials and Methods

No endangered or protected species or locations were involved in this study.

### Plant material

Twenty rubber clones (Table 1) and 191 genotypes from a biparental family were used in this study. Among the 20 clones, 13 were bred from the so-called Wickham population (i.e. the seeds historically transferred from Brazil to Kew Gardens in UK by Sir Wickham, in 1876), in Asia (GT1, PB217, PB254, PB260, RRIC100, RRIM600, RRIM901 and RRIM926) and Africa (IRCA18, IRCA19, IRCA303, IRCA41 and IRCA631). Seven clones (FDR4575, FDR5788, FDR5240, FDR5665, MDX607, MDX624 and CD1174) were bred in Latin America by crossing Wickham and native Amazonian parents. All clones were of the *Hevea brasiliensis* species except FDR5240 which was obtained by interspecific crossing (*H*. *brasiliensis* x *H*. *spruceana*).

**Table 1:** Rubber clones used in this study.

Clone^1^	Parentage (Female x Male)	Genetic group^2^	Origin
CD1174	AVROS1581 x MDF315	WA	
FDR4575	HARBEL68 x FDR18	WA	
FDR5240	HARBEL68 x TU45/525	WA	
FDR5665	HARBEL62 x MDX25	WA	Cirad Michelin Selection, Guatemala
FDR5788	HARBEL8 x MDF180	WA	
MDX607	AVROS1581 x MDF?	WA	
MDX624	AVROS1581 x MDF?	WA	
GT1	Primary clone	W	Gondang Tapen, Indonesia
IRCA18	PB5/51 x RRIM605	W	
IRCA19	PB5/51 x RRIM605	W	
IRCA303	GT1 x IR22	W	IRCA, Côte d’Ivoire
IRCA41	GT1 x PB5/51	W	
IRCA631	PB5/51 x RRIM707	W	
PB217	PB5/51 x PB6/9	W	
PB254	PB5/51 x PBS/78	W	Prang Besar, Malaysia
PB260	PB5/51 x PB49	W	
RRIC100	RRIC52 x PB86	W	RRIC, Sri Lanka.
RRIM600	TJIR1 x PB86	W	
RRIM901	PB5/51 x RRIM600	W	RRIM, Malaysia
RRIM926	PB5/51 x RRIM623	W	

^1^ All clones are of the *H*. *brasiliensis* species, except FDR5240 clone which is of an interspecific hybrid (*H*. *brasiliensis* x *H*. *spruceana*).

^2^ Genetic group: W (Wickham) genotypes originate from seeds historically transferred from Brazil to Kew Gardens (UK) by Sir Wickham in 1876 and then improved and multiplied in Asia and Africa; WA genotypes were created by crossing between W and A (native Amazonian) genotypes.

Eighteen clones (all clones listed in Table 1, except IRCA18 and IRCA631), grown at the SAPH (“Société Africaine de Plantations d’Hévéa”) plantation of Toupah, in Ivory Coast, were used for toxicity tests. Although initially selected for their good yield in rubber production, IRCA18 and IRCA631 were later found highly sensitive to the CLF disease in Africa and thus removed from the recommendation lists. They were no longer available at the SAPH-Toupah plantation.

Eight clones (IRCA41, GT1, RRIM600, RRIC100, PB217, IRCA18, PB260, and IRCA631), grown in a greenhouse (26/28°C night/day temperature and 40–60% humidity) at Cirad, Montpellier (France), were used for both inoculation and filtrate toxicity tests. In this experiment, they were chosen for their contrasted sensitivity to CLF in African plantations, with GT1 and IRCA41 the least sensitive, on the one hand, and IRCA18 and IRCA631 the more sensitive, on the other hand.

For genetic mapping and QTL analysis, the biparental population PB260 x RRIM600 was produced in Vietnam at the Lai-khê experimental station of the Rubber Research Institute of Vietnam, by hand pollination. The seeds were sent to Côte d’Ivoire, and 191 genotypes taken at random were multiplied by budding at the SOGB plantation (“Société de Grand-Béréby”). The male parent (RRIM600) is more tolerant to CLF than the female parent (PB260), in African plantations.

The clonal identities of all the plants were checked using a set of eight microsatellite (Simple Sequence Repeats, or SSR) markers.

### Fungal material

The ten *C*. *cassiicola* isolates used in this study (Table 2) were kindly provided by public Rubber Research Institutes (RRI), in Philippines (PRRI), Malaysia (RRIM), India (RRII), Sri-Lanka (RRISL) and Thailand (RRIT), as well as two privately owned rubber plantations in Cameroon (HEVECAM) and Côte d’Ivoire (SOGB). Isolates were collected from diseased rubber tree leaves, purified by single conidium isolation, and verified by sequencing of the PCR-amplified fragments of the ribosomal genes using ITS1 and ITS4 primers [27]. Their genetic group and toxin class were characterized by amplicon sequencing of four combined loci (ITS, actin, caa5, ga4) and the cassiicolin-encoding gene respectively, according to the typology proposed by Déon *et al*. [10]. The mycelium was cultivated on PDA medium (Potato Dextrose Agar), at 25°C in the dark. For long term conservation, mycelial plugs were kept at -80°C in 20% glycerol. For filtrate production, 100 ml of modified Czapeck medium [7] was inoculated with 6 mycelial plugs (5 mm diameter) from a 7-days-old culture on PDA medium at 25°C (photoperiod 12 h); the mycelium was grown in Czapeck medium for 21 days, at 25°C (photoperiod 12 h). The culture was filtered once through 0.45 μm Millipore membranes to remove most of the mycelium, then twice through 0.22 μm Millipore membranes. The last filtration was conducted under sterile laminar flow. The sterile filtrate was stored at 4°C, for up to three months.

**Table 2:** Treatments used in this study.

Blank treatments		
CZ	Culture medium	
Tox0	Water	
Purified cassiicolin Cas1^1^	Concentration	
Tox1	1 ng/μL	
Tox5	5 ng/μL	
Tox10	10 ng/μL	
*C*. *cassiicola* filtrates	Isolate type^2^	Geographical origin (provider)
CCP	C/Cas1	Philippines (PRRI)
CCAM3	C/Cas1	Cameroon (HEVECAM)
CLN16	A4/Cas0	Malaysia (RRIM)
CIND3	A4/Cas0	India (RRII)
CSRi5	A4/Cas0	Sri-Lanka (RRISL)
CCI6	A4/Cas0	Côte d’Ivoire (SOGB)
CCI13	A4/Cas0	Côte d’Ivoire (SOGB)
TSB1	B4/Cas5	Malaysia (RRIM)
CSB16	B4/Cas5	Malaysia (RRIM)
CTHA3	F1/Cas0	Thailand (RRIT)

^1^ Cassiicolin (isoform Cas1) purified from isolate CCP culture filtrate

^2^ Genetic group/toxin class, as characterized by Déon *et al*. [10]

PRRI, Philippines Rubber Research Institute; HEVECAM, Société Hévéa du Cameroun; RRIM, Rubber Research Institute of Malaysia; RRII, Rubber Research Institute of India; RRISL, Rubber Research Institute of Sri Lanka; SOGB, Société de Grand Béréby; RRIT, Rubber Research Institute of Thailand.

### Cassiicolin purification

Cassiicolin (isoform Cas1) was extracted from 1 L of culture filtrate from the isolate CCP, following the protocol in acidic conditions described previously [7]. The toxicity of the fractions was monitored by visual assessment of the symptoms (necrosis) on PB260 detached leaflets. The purified toxin was conserved dried (lyophilized) for years or diluted in water, at 4°C, for at least three months.

### Toxicity test

The various treatments assessed for toxicity are listed in Table 2. They include crude culture filtrates from 10 *C*. *cassiicola* isolates of various types and geographical origins, the cassiicolin isoform Cas1 purified from isolate CCP, at three concentrations (Tox1, Tox5, Tox10), and two blank treatments (water and Czapeck culture medium). The phytotoxicity was assessed on detached rubber tree leaflets by conductivity measurement of the induced electrolyte leakage, as conducted in several ecophysiological studies on rice [28], tomato [29] or rubber [30]. Healthy leaves collected in the morphogenetic stage C (limp, brownish to green) [31] were placed in large square Petri dishes, on wet paper. Two drops (15 μL each) of each treatment solution (Table 2) were applied on the abaxial surface of each leaflet after gentle scraping of the lower epidermis (1 mm^2^). The Petri dishes were kept in the dark at 26°C for 48 h. Then two leaf disks (2.2 cm^2^) per leaflet were punched out using a cork borer, at the drop application point, and soaked in 5 mL of autoclaved distilled water in glass tubes, for 24 h, in the dark, at 26°C.

The conductivity of the solution (C_1_, in μS/cm), indicating the response of the rubber leaf sample to the treatment, was measured using the conductivity meter 3310 (WTW, Weilheim, Germany) and the conductivity cell TetraCon^R^ 325. Then the tubes containing the solutions with the two leaf disks were autoclaved for 15 min at 121°C and 212 kPa. After autoclaving, the conductivity of the solution was measured again (C_2_, in μS/cm). C_2_ appeared to be close to the maximum conductivity value measurable for each sample: it was checked that repeating the autoclave process one more time did not increase the C_2_ value. The percentage of electrolyte leakage induced by each treatment (EL%) was calculated as (C_1_/C_2_)*100. The normalization of C_1_ by C_2_ aims at correcting the leaf samples’ intrinsic variability in their capacity to release electrolytes, in the conditions of our test.

At least three biological replicates were performed for each clone/treatment combination. Each biological repeat was conducted by a single operator in order to analyze the operator-dependent variations (such as variations in the leaf epidermis scraping) as a block effect. The sensitivity of a clone to a specific treatment was estimated to be low for an average %FE value below 30, moderate between 30 and 60, and high above 60.

### Inoculation

Inoculations were performed with two *C*. *cassiicola* isolates (CCI13 and CCP) on detached leaflets of eight clones (GT1, PB217, PB260, RRIC100, RRIM600, IRCA18, IRCA41 and IRCA631) maintained in greenhouse at Montpellier. Isolate CCP was chosen for its high aggressiveness [6,10]; CCI13 was a newly sampled local isolate from Ivory Coast, chosen among the widely distributed type A4/Cas0 isolates. The isolates were cultivated on PDA medium at 25°C for four days in the dark, then three days under alternate light to stimulate sporulation, as adapted from Chee *et al* [32]. The conidia were collected and resuspended in sterile water supplemented with 0.02% Tween20 at a concentration of 10^4^ conidia mL^-1^. Six to eight drops of each conidial suspension (20 μL per drop) were applied to the abaxial surface of detached rubber tree leaflets in the developmental stage C. The leaflets were maintained in a moist environment at 25°C for 24 h in the dark and then for 72 h under alternate light (photoperiod 12 h). The necrotic area was measured by ImageJ software from the photo taken four days post-inoculation.

### Genotyping with SSR markers

The 191 individuals from the biparental family PB260 x RRIM600 were genotyped with SSR (Simple Sequence Repeats, or microsatellites) molecular markers from rubber tree, including 300 markers from SSR-enriched genomic libraries and 92 markers from SSR-containing EST (Expressed Sequence Tag) or SSH (Suppressive Subtractive Hybridization) cDNA sequences [33–35]. For each SSR marker, one of the two framing primers was tailed with a short M13 DNA sequence in order to attach a fluorochrome during the preparation of the PCR mix. All primer sequences are available from previous publications [34,35]. Genomic DNA from each individual was extracted from fresh leaves of young plants according to Risterucci *et al*. [36] and quantified by fluorescence (Fluorochrome Hoechst 33258). Each PCR amplification was performed in a 10 μL reaction solution containing 25 ng of DNA, 2 mM MgCl_2_, 200 μM dNTP, 1.0 U Taq DNA polymerase, 1.0 x PCR buffer, and 0.2 μM of each primer, with molecular biology grade water up to 10 μL. The program for PCR was denaturation at 94°C for 4 min, followed by 10 cycles of denaturation (94°C for 45 sec), annealing (55°C for 1 min for the first cycle), and elongation of the second strands (72°C for 75 sec), reducing the annealing temperature by 0.5°C per cycle starting at 55.0°C and down to 50.5°C; then 25 cycles were carried out with an annealing temperature of 50°C (94°C for 45 sec for denaturation and 72°C for 1 min for elongation). A final elongation step was performed at 72°C for 5 min. Amplicons were migrated by capillary electrophoresis on ABI3500xL equipment and the migration images analyzed by Genemapper software (Applied Biosystems 2007).

### Statistical analysis, linkage map construction and QTL mapping

Statistical analyses were performed with software R (version 3.0.3). Analyses of variance were performed with linear models using Rcmdr and lme4 packages. The correlation between variables was calculated by Pearson correlation coefficient using Rcmdr package.

In the study of the responses of 18 clones (all clones in Table 1, except IRCA18 and IRCA631) to 15 treatments (Table 2), the blocks, the clones, and the treatments were considered as fixed effects; the mean values were calculated by Lsmeans package, and the significance of differences was tested using Student-Newman-Keuls (SNK) test (at risk α = 0.05, unless specified). On the matrix data of the global effects of the clone x treatment combination, a double clustering was carried out by use of the function heatmap.2 of gplots package, based on Euclidean distances and on hierarchical clustering using the “complete linkage” aggregation method.

Broad-sense heritability estimation (H^2^) for the trait of sensitivity to the different toxinic treatments was calculated using the following formula: H^2^ = σ^2^_g_/(σ^2^_g_ + σ^2^_e_/n), where n is the mean number of replicates per genotype, σ^2^_g_ is the estimated genetic variance (*i*.*e*. inter-genotype variance), and σ^2^_e_ the residual error variance.

Linkage map construction was performed with the software JoinMap 4.1 [37] using a regression mapping algorithm [38] and the genetic distance function of Kosambi. QTL analysis was performed using the software MapQTL 6.0 [39]. A permutation test [40] was applied with 1000 rounds to determine the significance threshold of the LOD score (logarithm (base 10) of odds, [41]) for QTL detection. The Interval Mapping (IM) method [42] was initially implemented, then the markers nearest to the first detected QTL were taken as co-factors so as to run a multiple-QTL (MQM) analysis [43,44] with the aim to detect other QTL of smaller importance. Each QTL was characterized by its LOD score (significance level), its most probable position on the genome, its percentage of explanation of the phenotypic variance (R²), and the effects of the parental alleles involved in its expression.

### Assessment of allelic effects on the QTL expressions

For any individual of the population, each QTL expression results from the addition of the three following effects: effect of the allele from the female parent, effect of the allele from the male parent, and interaction between both alleles. These effects can be estimated at the position of the nearest marker in the neighborhood of the most probable QTL position, where the allelic constitutions of the individuals of the population are completely known. If the segregation ratio of the marker is 1:1:1:1, the variance analysis of the phenotypic data of one trait can provide an estimation of the parental effects (two alleles per parent equivalent to two levels of treatment), and of the interaction between both parents for this trait. For a marker with a segregation ratio 1:1 with only one polymorphic parent, only the effect of this parent can be tested. This method was used in order to assess the allelic effects of the QTL detected for the different toxicity tests applied to the population.

## Results

### Methodological validation of the toxicity test

**Fig 1:**
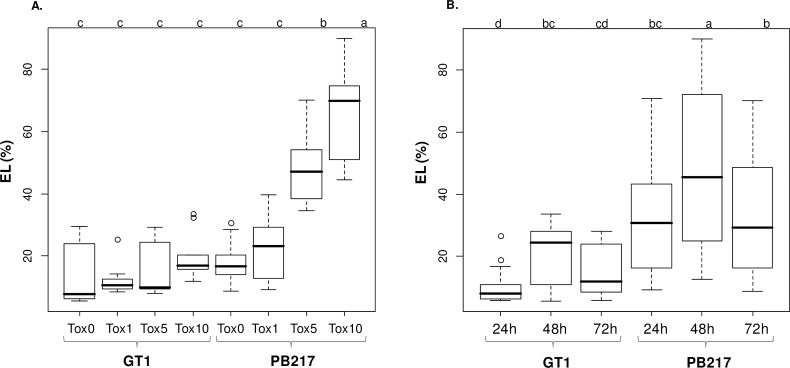
**Effect of toxin concentration (A) and incubation duration (B).** Distribution of the electrolyte leakage data (EL%) measured from leaf tissues treated with the purified toxin Cas1 at 0, 1, 5 and 10 ng/μL (Tox0, Tox1, Tox5 and Tox10 respectively) and three incubation durations (24, 48 and 72 h), on the clones GT1 (tolerant) and PB217 (susceptible). Three biological repeats were performed for each condition. Top letters indicate the significance of the differences between treatments (SNK test, risk α = 0.05).

The toxicity test was first validated using the purified toxin Cas1 (cassiicolin), secreted by the *C*. *cassiicola* isolate CCP, on the rubber clones GT1 and PB217 known to be respectively tolerant and susceptible to this isolate [6,9,45]. Under these conditions, the test appeared to be both quantitative, with EL% values proportional to the concentration in toxin (Fig 1A), and qualitative, with EL% values in good coherence with the known susceptibility of the clones (Fig 1A and 1B). On the same data set, the optimum duration for the incubation of the toxin on the leaf tissue was estimated to be 48 h, for both clones (Fig 1B), with high median EL% values and a strong dispersion of the data reflecting the various responses to the four toxin concentrations. A 24 h incubation appeared insufficient, especially for tolerant clones such as GT1. With a longer incubation (72 h), the dispersion and maximum EL% values were lower, suggesting that some of the released electrolytes were no longer measurable, having probably passed through the cuticle of the upper epidermis, degraded by the prolonged action of the toxin. A 48 h incubation is therefore recommended for routine analysis.

**Fig 2:**
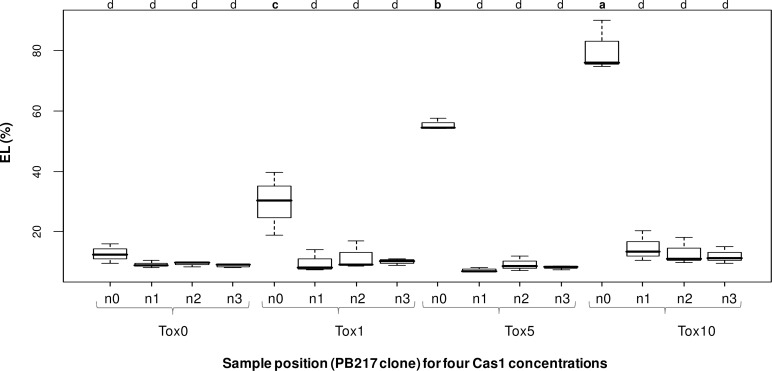
Extent of toxin diffusion through the leaf tissue. Distribution of the electrolyte leakage data (EL%) measured from detached leaves (susceptible clone PB217) treated with purified cassiicolin Cas1 at 0, 1, 5 or 10 ng/μL (Tox0, Tox1, Tox5, Tox10 respectively) depending on the distance from the toxin application point. After incubation for 48h, leaf pieces (2.2 cm^2^) were cut out at the application point (n0) or at adjacent positions of increasing distances (2, 4, 6 cm, corresponding to positions n1, n2 and n3 respectively) from n0. For each condition, three biological repeats were performed. Top letters indicate the significance of differences between treatments (SNK test, risk α = 0.05).

As shown in Fig 2, the EL% values measured at increasing distance (positions n1, n2 and n3) from the toxin application point (n0) were not significantly different from the untreated control, whatever the concentration in toxin, suggesting a limited diffusion of the toxin through the leaf tissue. Only the leaf pieces collected at the application point (n0) gave EL% values in good correlation with the concentration in applied toxin, *i*.*e*. representative of the leaf sensitivity to the toxin.

**Fig 3:**
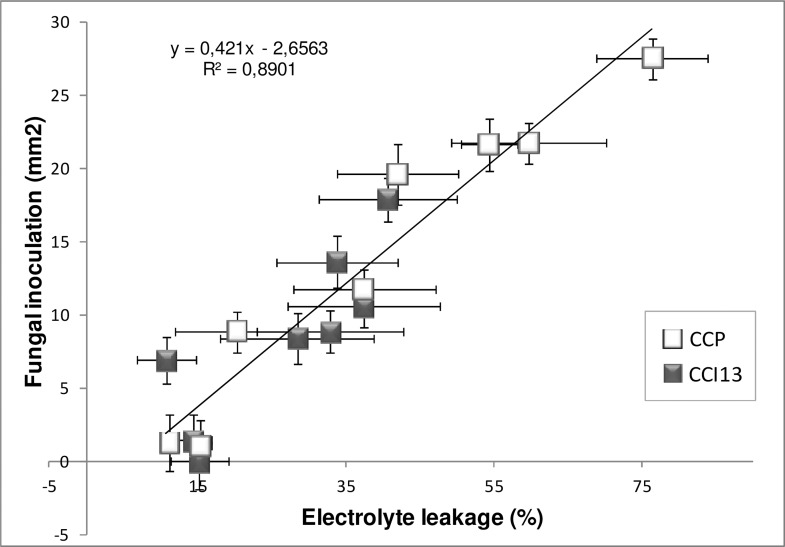
Correlation between the sensitivity of rubber clones to culture filtrates and their susceptibility to spore inoculation. Eight clones (GT1, PB217, PB260, RRIC100, RRIM600, IRCA18, IRCA41 and IRCA631) were treated with culture filtrates or spore suspensions from two isolates (CCP and CCI13), on detached leaves. Sensitivity to the culture filtrates was expressed as the induced electrolyte leakage EL%. Susceptibility in response to spore inoculation was expressed as the surface of leaf necrosis. The error bars are standard errors over three biological repeats. See S1 Table for details.

The toxicity test was finally validated by comparing the response of eight clones to either the culture filtrate (fungal exudates) or the fungus itself inoculated as conidial suspension, for two isolates, CCP and CCI13 (Fig 3 and S1 Table). Overall, a positive correlation was observed between the two methods: among the 16 couples of data, 89% (R²) of the variation of the leaf necrosis areas due to the conidial inoculations was predicted by the results from the toxicity tests, which is equivalent to a correlation coefficient of 0.94 (significant threshold r = 0.62 for df = 14 at risk α = 0.01) (Fig 3). Considering each isolate separately, the coefficients of Pearson correlation between the two variables (EL% and necrosis area) were 0.97 and 0.84, for CCP and CCI13 respectively (S2 Table).

### Phenotyping of 18 clones for their sensitivity to *C*. *cassiicola* exudates, by electrolyte leakage measurement (ELM)

Eighteen clones were tested for their sensitivity to 15 treatments (Table 2): two blank treatments (water and the culture medium CZ), the purified toxin Cas1 at three concentrations, or crude culture filtrates from various *C*. *cassiicola* strains (seven Asian and three African), representative of four types (genetic group/toxin class) based on the previously established typology [10]. Sensitivity was monitored by EL% measurement (Table 3).

**Table 3:** Electrolyte leakage response (EL%) of 18 clones to *C*. *cassiicola* exudates (15 treatments).

TreatmentClone	Tox0Water	CzCulture medium	Tox1Cas1 (1ng/μL)	CTHA3F1/Cas0	CLN16A4/Cas0	TSB1B4/Cas5	CSB16B4/Cas5	CIND3A4/Cas0	Tox5Cas1 (5ng/μL)	CCAM3C/Cas1	CCPC/Cas1	CSRI5A4/Cas0	CCI13A4/Cas0	Tox10Cas1 (10ng/μL)	CCI6A4/Cas0	*Mean per clone*
FDR5240	12.0	13.8	18.1	19.9	19.0	25.4	17.5	17.8	30.3	21.3	14.1	22.8	21.8	25.2	37.0	*21*.*1*^*fg*^
IRCA41	13.2	11.2	19.7	21.9	25.6	16.7	22.2	29.0	35.0	38.6	33.2	12.6	34.6	43.1	15.2	*24*.*8*^*fg*^
RRIM600	20.7	17.5	20.3	22.4	29.1	29.5	34.2	34.6	28.9	41.0	29.9	42.4	42.1	28.8	35.9	*30*.*5*^*f*^
FDR5665	17.3	21.0	30.2	19.5	36.7	29.7	25.1	47.1	27.6	42.4	47.7	48.5	55.6	43.7	63.7	*37*.*1*^*e*^
MDX624	21.2	20.4	16.2	21.2	28.4	26.4	22.8	40.8	38.2	54.7	44.9	55.4	61.5	44.4	86.8	*38*.*9*^*de*^
CD1174	23.6	23.4	17.2	22.4	29.1	27.1	35.3	35.7	21.8	55.2	43.3	73.1	78.0	40.6	78.1	*40*.*3*^*cd*^
RRIC100	19.1	18.3	25.2	26.7	36.5	30.9	48.3	38.8	54.3	55.2	36.5	60.2	49.0	82.8	44.5	*41*.*8*^*cd*^
MDX607	24.2	27.2	37.2	44.7	37.8	21.4	33.8	36.7	45.6	43.1	48.7	61.6	41.7	79.2	74.5	*43*.*8*^*cd*^
IRCA303	29.5	39.0	44.4	64.4	29.0	49.9	51.2	40.0	33.2	41.6	43.5	47.2	45.2	46.3	74.2	*45*.*2*^*c*^
PB217	18.7	24.9	45.3	44.9	41.5	46.8	40.7	42.7	63.9	44.5	71.1	41.0	42.3	76.3	42.5	*45*.*8*^*c*^
IRCA19	15.3	21.0	52.7	18.4	28.7	23.1	34.0	34.7	71.9	53.7	81.7	49.6	51.5	89.3	62.8	*45*.*9*^*c*^
PB254	21.8	25.0	55.3	38.1	32.7	44.1	61.0	47.1	87.1	51.0	84.1	45.1	56.6	88.2	56.6	*52*.*9*^*b*^
GT1	46.0	59.6	37.2	54.6	46.2	49.7	61.4	60.9	47.1	57.2	40.1	63.0	66.0	61.2	58.2	*53*.*9*^*b*^
RRIM901	16.8	27.4	27.9	36.8	54.1	39.4	31.9	39.9	68.7	78.8	65.5	94.5	80.0	64.1	90.5	*54*.*4*^*b*^
FDR5788	17.8	16.3	29.4	28.4	38.6	43.0	44.3	41.7	69.7	86.5	82.1	69.6	78.2	86.8	90.4	*54*.*9*^*b*^
FDR4575	37.4	42.6	45.3	43.0	45.0	55.7	50.5	53.5	54.4	69.4	72.1	92.5	95.5	72.6	93.3	*61*.*5*^*a*^
RRIM926	13.8	19.7	42.3	47.1	43.5	53.7	48.4	68.9	78.3	84.1	89.4	90.8	93.5	77.4	93.7	*63*.*0*^*a*^
PB260	13.9	44.2	51.6	43.1	53.5	62.0	69.6	63.3	63.2	81.4	80.7	85.0	87.6	94.6	88.1	*65*.*5*^*a*^
*Mean per treatment*	*21*.*2*^*h*^	*26*.*3*^*h*^	*34*.*2*^*g*^	*34*.*3*^*g*^	*36*.*4*^*fg*^	*36*.*5*^*efg*^	*40*.*7*^*ef*^	*43*.*0*^*e*^	*51*.*1*^*d*^	*55*.*6*^*cd*^	*56*.*0*^*cd*^	*58*.*6*^*bc*^	*60*.*0*^*bc*^	*63*.*6*^*ab*^	*65*.*9*^*a*^	

The 15 treatments were the purified cassiicolin Cas1 at 1, 5 and 10 ng/μL (Tox1, Tox5, and Tox10 respectively, grey columns), filtrates from 10 isolates (with the genetic group/toxin class indicated under the isolate name) and two blank treatments, *i*.*e*. water (Tox0) and the culture medium Cz. For each clone x treatment combination, the EL% value is the mean from at least three biological replicates. The significance of the clone and treatment effects (superscript letters associated with the mean per clone and mean per treatments values) was estimated separately for each effect using the SNK test (risk α = 0.05).

Among the 270 clone x treatment combinations, EL% varied from 11.2 to 95.5%, with the general mean equal to 45.6% (Table 3). The clones were ranked based on their mean response to the 15 treatments, from the more sensitive (PB260, RRIM926 and FDR4575) to the less sensitive (FDR5240, IRCA41, RRIM600). The treatments were ranked based on their mean effect on the 18 clones, from the least aggressive (Tox0 and Cz) to the most aggressive (CCI6). When focusing on the responses induced by the purified cassiicolin Cas1 (grey columns in Table 3), we could observe that the mean intensity of the response was proportional to toxin concentration. The toxicity measured for the CCP filtrate (from which cassiicolin was purified) was intermediate between the responses obtained with 5 and 10 ng/μL of purified toxin.

**Fig 4:**
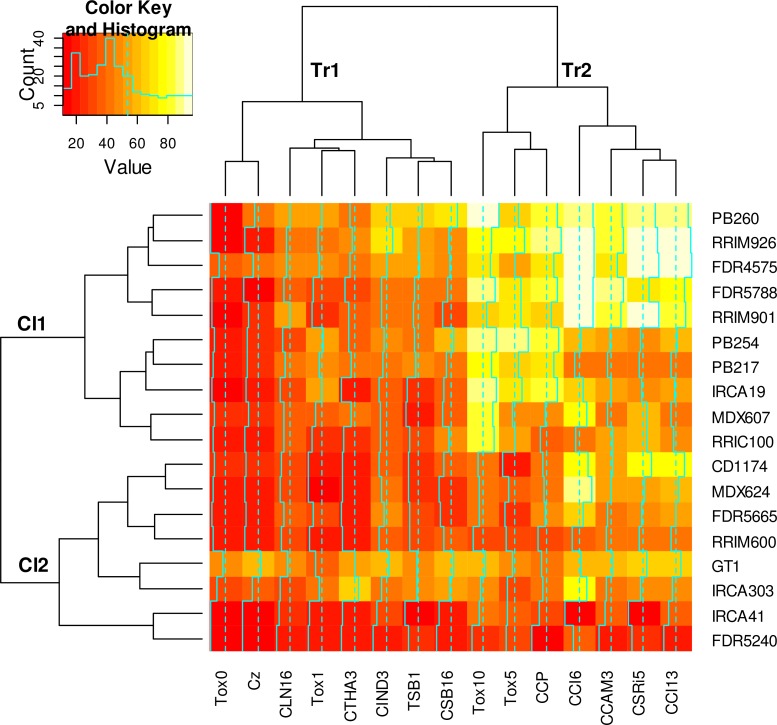
Electrolyte leakage response of 18 clones to 15 treatments: matrix of the EL% data from Table 3 and hierarchical clustering. Hierarchical clustering of the 18 clones (Cl) and 15 treatments (Tr) was performed by the “complete linkage” aggregation method from Euclidian distances. Color key from dark to light colors indicates low to high EL% values.

In the variance analysis applied to the whole data set (Table 4), the effects due to the clones and the treatments as well as the clone x treatment interaction were highly significant (p<0.001). The sums of squares of these three factors (clone, treatment, interaction) were of the same magnitude, thus indicating that they were roughly equivalent in their contributions to the explanation of the data variations. The statistical model explained R² = 75% of the total variation. Fig 4 shows the heatmap response for the whole EL% data set, and the hierarchical clustering (by pairwise dissimilarities) of the clones (Cl), on left side, and treatments (Tr), on top. Concerning the treatments, two main clusters were identified. Cluster Tr1 corresponds to the eight treatments of low mean toxicity (Tox0, Cz, CLN16, Tox1, CTHA3, CIND3, TSB1, and CSB16), with one sub-cluster corresponding to the two blank treatments Tox0 and Cz. Cluster Tr2 corresponds to seven treatments of high mean toxicity and is divided into two sub-clusters: CCP and the related Tox5 and Tox10 treatments on the one hand, and CCI6, CCAM3, CSRI5, and CCI13 on the other hand. Concerning the clones, two main clusters were also identified. Cluster Cl1 corresponds to 10 clones highly sensitive to Tox10. Within Cl1, five clones (PB260, RRIM926, FDR4575, FDR5788 and RRIM901) were highly sensitive to all Tr2 treatments, while the responses of the others clones were more diverse: RRIC100 was highly sensitive to Tox10 only; MDX607 was highly sensitive to Tox10 and CCI6; and the other three (PB254, PB217, IRCA19) were highly sensitive to Tox10 (and related motifs Tox5 and CCP) but clearly less to the other Tr2 treatments. Cluster Cl2 corresponds to eight clones of variable sensitivity but clearly less sensitive to Tox10, Tox5 and CCP, compared to the clones in Cl1. Two clones in Cl2 (MDX624 and IRCA303) were highly sensitive to CCI6.

**Table 4:** ANOVA of the EL% response of 18 clones to 15 treatments.

Component	Df	Sum Sq	Mean Sq	F value	Pr (>F)
block	2	2961	148.5	6.8	0.0012**
clone	17	125258	7368.1	33.8	< 2.2e-16***
motif	14	149761	10697.2	49.1	< 2.2e-16***
clone:motif	238	121375	510	2.3	< 2.2e-16***
Residuals	614	133823	218.0		

The 15 motifs were the purified cassiicolin Cas1 at 1, 5 and 10 ng/μL (Tox1, Tox5 and Tox10 respectively), 10 filtrates and two blank treatments, water (Tox0) and the culture medium Cz. R^2^ = 0.75;

‘**’ p<0.01

‘***’ p<0.001

### Identification of QTL associated with the response of rubber tree to *C*. *cassiicola* exudates

**Fig 5:**
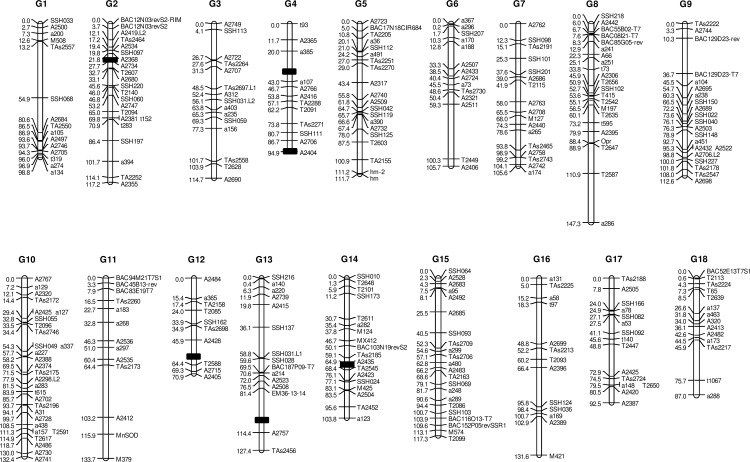
Genetic linkage map of rubber tree and QTL associated with the sensitivity/insensitivity to *C*. *cassiicola* exudates. The map was obtained from 191 F1 progenies of the PB260 x RRIM600 family. All the markers are SSR markers. The 18 linkage groups (g1-g18) correspond to the 18 chromosomes of the haploid rubber tree genome. Black boxes indicate the position of the QTL.

A genetic linkage map was built from the F1 progeny of the PB260 x RRIM600 cross using microsatellite markers (SSR) exclusively. A subset of 191 progenies planted in Côte d’Ivoire was screened with 392 SSR markers, among which 306 segregated. Table 5 shows the five possible classes of segregation with the number of markers in each group. The Mendelian segregation ratios were in accordance with a diploid inheritance of most markers, with only seven markers (2%) showing some segregation distortion. Eighteen linkage groups were found, corresponding to the 18 chromosomes of the haploid rubber tree genome (Fig 5). The overall coverage was of 2005 cM, with an average intermarker distance of 6.6 cM.

**Table 5:** Number of SSR markers segregating in the F1 progeny of the PB260 x RRIM600 family, for each segregation type.

Type of segregation	Genotypes in the progeny	Mendelian segregation ratio	Number of segregated SSR markers
<abxcd>	ac, ad, bc, bd	1: 1: 1: 1	38
<efxeg>	ee, eg, ef, eg	1: 1: 1: 1	81
<hkxhk>	hh, hk, kk	1: 2: 1	36
<lmxll>	lm, ll	1 : 1	94
<nnxnp>	nn, np	1 : 1	57
*Total markers*			*306*

The 5 types of segregation, as defined by Ritter et al. [46] for crosses between heterozygous parents are coded according to JoinMap software instructions [37].

**Fig 6:**
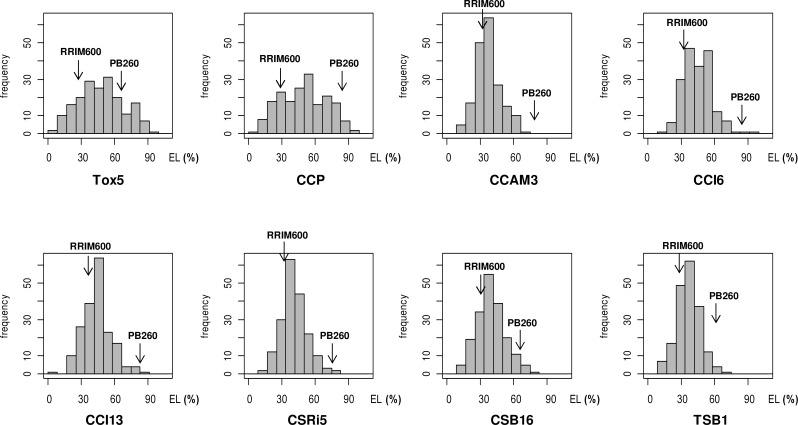
Distribution of the electrolyte leakage (EL%) data of the F1 progenies, for each treatment. The treatments were the purified toxin Cas1 at 5 ng/μL (Tox5) and culture filtrates from seven *C*. *cassiicola* isolates. Parental EL% values (PB260 and RRIM600) are indicated by arrows. Shapiro-Wilk test at risk α = 0.05.

Progenies were phenotyped using the ELM method for their sensitivity to the purified toxin Cas1 at 5 ng/μL (Tox5) or to seven *C*. *cassiicola* culture filtrates (CCP, CCAM3, CCI6, CCI13, CSRI5, CSB16, and TSB1) of various geographic origins and three genetic groups. The EL% values of the F1 progenies for each treatment followed normal distributions (Fig 6), indicating a quantitative response to the eight treatments. The EL% values of the parental clones were contrasted, with the maternal clone PB260 more sensitive than the paternal clone RRIM600, whatever the treatment.

Analysis of the Pearson correlation coefficients between the various treatments for their effect on the progeny (Table 6) revealed a highly significant positive correlation between CCP and Tox5. All other filtrates were strongly correlated to each other but not to CCP and Tox5 (or only moderately so). The blanks (water and Cz) were significantly correlated to each other but not to any other treatment.

**Table 6:** Pearson correlation coefficients between treatments calculated from the electrolyte leakage values (EL%) on the F1 progeny.

Treatment	Type of isolate	Tox5	CCP	CCAM3	CCI6	CCI13	CSRI5	CSB16	TSB1	Cz
CCP	C/Cas1	0.86^***^								
CCAM3	C/Cas1	0.18	0.25^*^							
CCI6	A4/Cas0	0.23	0.30^*^	0.70^***^						
CCI13	A4/Cas0	0.23	0.28^*^	0.72^***^	0.81^***^					
CSRI5	A4/Cas0	0.25^*^	0.30^*^	0.74^***^	0.75^***^	0.78^***^				
CSB16	B4/Cas5	0.09	0.13	0.64^***^	0.70^***^	0.70^***^	0.67^***^			
TSB1	B4/Cas5	0.20	0.19	0.64^***^	0.67^***^	0.63^***^	0.67^***^	0.62^***^		
Cz	-	0.14	0.17	0.03	-0.01	0.03	-0.03	-0.03	0.04	
Water	-	0.13	0.16	0.14	0.10	0.10	0.10	0.03	0.13	0.51^***^

The treatments were the purified toxin Cas1 at 5 ng/μL (Tox5) and culture filtrates from seven *C*. *cassiicola* isolates. Blank treatments are water and the culture medium Cz. Significance level of the correlation coefficients

‘*’ p<0.05

‘**’ p<0.01

‘***’ p<0.001

QTL associated with the response to seven filtrates or to the purified toxin Cas1 were detected from the PB260 x RRIM600 F1 progeny and mapped on the genetic linkage map (Table 7 and Fig 5). The estimated broad-sense heritability (H^2^) varied between 0.44 and 0.79 for the filtrates and the toxin, with the highest values for Tox5, CCP and CSB16 (H^2^ > 0.70). For the two blank treatments (water and Cz), heritability was very low (between 0.007 and 0.18).

**Table 7:** Broad-sense heritability (H^2^), QTL and parental allelic effects (%) calculated from the response (EL%) of the PB260 x RRIM600 F1 family to *C*. *cassiicola* exudates.

									Parental allelic effect (%)^e^		
Treatment	Type of isolate	H^2^	QTL^a^	LOD peak	R^2^ (%)^b^	Closest marker^c^	Accession^d^	Segregation ratio	PB260	RRIM600	Interaction
Tox5	C/Cas1	0.78	g2-26	7.5	17	A2734	AY486840	1:1:1:1	10***	6***	-
			g4-95	4.9	11	A2404	AY486695	1:1:1:1	11***	-	-
CCP	C/Cas1	0.78	g2-26	5.4	12	A2734	AY486840	1:1:1:1	10***	3*	-
			g4-95	5.3	12	A2404	AY486695	1:1:1:1	12***	-	-
CCAM3	C/Cas1	0.58	g12-53	5.9	13	A2428 or T2588	AY486714 or AY486764	1:1	-	-	-
			g13-102	5.0	11	A2757	AY486813	1:1:1:1	-	-	8***
CCI6	A4/Cas0	0.49	g4-32	4.9	11	a107	AY486864	1:1:1:1	-	-	8***
CCI13	A4/Cas0	0.44	g4-32	6.0	14	a107	AY486864	1:1:1:1	-	-	10***
CSRI5	A4/Cas0	0.55	g4-32	5.4	12	a107	AY486864	1:1:1:1	-	-	9***
			g13-102	4.8	11	A2757	AY486813	1:1:1:1	-	-	8***
CSB16	B4/Cas5	0.79	g13-102	5.6	13	A2757	AY486813	1:1:1:1	-	-	7***
TSB1	B4/Cas5	0.51	g14-64	7.0	16	A2435	AY486718	1:1:1:1	-	18***	3*
*Water*	*-*	*0*.*007*	-			-		-	-	-	-
*Cz*	*-*	*0*.*18*	-			-		-	-	-	-

The PB260 x RRIM600 F1 family was treated with the purified toxin Cas1 at 5 ng/μL (Tox5), culture filtrates from seven *C*. *cassiicola* isolates and two blank treatments (water and the culture medium Cz). The measured response was the induced electrolyte leakage (EL%). QTL, calculated by Interval Mapping method, were significant at the LOD threshold of 4.2 (risk α = 0.05).

^a^ QTL are named by the number of the linkage group (g) on which they are located and their position (Fig 5).

^b^ R^2^ is the percentage of explained phenotypic variance

^c^ Closest SSR marker from the LOD peak

^d^ Accession number of the markers in EMBL databank (https://www.ncbi.nlm.nih.gov/genbank)

^e^ Significance level of the parental allelic effect

‘*’ p<0.05

‘**’ p<0.01

‘***’ p<0.001

A total of six QTL were identified, with LOD scores varying from 4.8 to 7.5. For the response to Tox5 and CCP, two common QTL were detected at positions g2-26 and g4-95, with percentages of explained phenotypic variance (R^2^) varying from 11 to 17%. For the three filtrates of isolate type A4/Cas0 (CCI6, CCI13 and CSRI5), a common QTL was detected at position g4-32, explaining 11, 14 and 12% respectively of the phenotypic variance. For filtrates CCAM3, CSB16 and CSRI5, of three different isolate types, a common QTL was detected at position g13-102, explaining respectively 11, 13 and 11% of the phenotypic variance. A second QTL was detected for filtrate CCAM3, at position g12-53, explaining 13% of the phenotypic variance. Finally, a single QTL was detected for filtrate TSB1, at position g14-64, which explained 16% of phenotypic variance.

The allelic effects on QTL expression were assessed (Table 7). For the two QTL g2-26 and g4-95 associated with the Cas1 toxin and CCP filtrate, the allelic effects were tested at the position of the markers A2734 and A2404 respectively. In both cases, the effect of the female parent, due to the difference between both alleles of this parent without any detectable interaction, was highly significant and explained the larger part (21 and 22% for Tox5 and CCP respectively) of the phenotypic variation expressed by the QTL. The effect of the male parent was also significant for QTL g2-26 but it explained a lesser part (3%) of the phenotypic variation. There was no significant interaction between alleles from both parents. For the QTL g12-53 associated with CCAM3, located between the markers A2428 and T2588, no parental effect could be detected. For the QTL g4-32 (associated with CCI6, CCI13, and CSRI5) and g13-102 (associated with CCAM3, CSRI5, and CSB16), there was no parental effect but a highly significant effect of interaction between alleles from both parents explaining the major part of the phenotypic variation for this trait. For the QTL g14-64 associated with TSB1, there was a highly significant effect of the male parent and a significant effect of the interaction.

## Discussion

### Development and validation of a phenotyping method

We have developed an indirect phenotyping method consisting in testing the sensitivity of rubber tree clones to *C*. *cassiicola* exudates, as a parameter of their global susceptibility to that fungus infection. This method is derived from the leaf puncture assay described by Breton *et al*. [6]: exudates were applied as crude culture filtrates or as purified toxin (Cas1) in case of the isolate CCP, on detached rubber tree leaves. However, instead of measuring the surface of induced leaf necrosis, the impact of the exudates was quantified by Electrolyte Leakage Measurement (ELM).

The ELM method was found to be more sensitive and accurate than the simple measurement of visual symptoms. In fact, of all the filtrates tested in this study, only CCP as well as the purified Cas1 toxin extracted from CCP gave clear visual symptoms (necrosis with darkening of the veins) as early as 48 h after application. With all the other filtrates, only the conductivity method was sensitive enough to reveal some variability among the clones within this timeframe. Therefore, the ELM method is clearly more appropriate than the visual assessment for quantifying the early cellular damages induced by fungal exudates. Another test, called the leaf-wilting test, was previously used in assessing the effect of *C*. *cassiicola* toxins on detached leaves, by measuring the induced water losses: detached leaflets were placed in a glass container with the petiole tip immersed in toxin solution or culture filtrate; the leaf wilting intensity was quantified after two days as the fresh weight out of dry weight ratio, in percent of a control [6,26]. However, we found this method more prone to variability, probably owing to the risk of external contamination when used in a tropical environment, and more costly in filtrate or purified toxin.

The toxicity test based on the ELM method was found to be quantitative. Indeed, the EL% response of 18 clones to three concentrations of purified cassiicolin Cas1 (Table 3, grey columns) was significantly correlated to toxin concentration (S1 Fig), thus confirming the observation initially made on two clones (Fig 1A and 1B). The test was able to reveal a large phenotypic diversity among clones and isolates, with a significant interaction effect added to these two principal effects. Owing to its practicality and rapidity, the test enables one to handle a large number of individuals, and as a consequence, it is a valuable tool for genetic studies and QTL identification. Because it uses purified toxin or filter-sterilized fungal culture instead of living cultures of the fungus, it allows for the exploration of the highly diverse *C*. *cassiicola* species with no threat to the environment.

We found a high specific positive correlation between the sensitivity of eight clones to culture filtrates from two virulent strains, CCP and CCI13, measured by the ELM method, and their susceptibility to the fungal infection, tested by conidial inoculation, on detached leaves. In a previous experiment, using the leaf wilting assay to evaluate the susceptibility of 51 clones to CLF disease, Breton *et al*. also found a good correlation between sensitivity to the filtrate and susceptibility to fungal infection [6]. However, this was tested with a single isolate (CCP). Out of seven clones (GT1, IRCA41, RRIM600, PB217, RRIC100, PB260 and IRCA631) in common between Breton’s study (leaf wilting test) and our study (ELM method, S1 Table and Table 3) and tested for the same filtrate (CCP), three were found to be tolerant (GT1, IRCA41 and RRIM600) and three were sensitive (PB217, PB260 and IRCA631), in both studies. The case of RRIC100 is ambiguous since it was noted as sensitive to CCP filtrate with the leaf wilting test, but not (or weakly) sensitive with the ELM test (S1 Table and Table 3 respectively).

Since these tests were conducted on detached rubber tree leaves, they can only account for early interaction events such as the capacity of the fungus to penetrate the cells, or the recognition of fungal effectors by plant receptors as the triggering signal for disease or resistance reactions. These early events are decisive for disease occurrence. Nevertheless, late defense mechanisms able to limit the progress of the disease may also occur. Phenotyping by inoculation on whole plants would be very informative but more time consuming and risky to handle in areas of rubber production, unless limited to local isolates.

To further evaluate the potential of this *in vitro* test in predicting rubber clones susceptibility to CLF, one should investigate whether sensitivity to culture filtrates or purified fungal toxin correlates with field susceptibility to the disease. In our study, the classification of eighteen clones (Fig 4) is partially consistent with their known susceptibility to CLF disease in African plantations. During the CLF epidemic in Cameroon in 1988–1992, PB260 was observed to be very susceptible, whereas IRCA19 was tolerant. In our study PB260 was sensitive to both CCP and African filtrates (strains CCI6 and CCI13), while IRCA19 was susceptible to CCP filtrate, yet less susceptible to the African filtrates, which may explain why this clone was observed to be tolerant in Cameroon. In 2003, a clone list describing the susceptibility/tolerance to CLF in various countries was published [47]. The clone GT1 was noted as highly sensitive in Malaysia, Indonesia and Thailand but relatively resistant in Africa and Sri Lanka; conversely, the clone PB260 was noted as tolerant in Asia but very sensitive in Africa. This had raised the hypothesis of the existence of various pathotypes among *C*. *cassiicola*. We thus investigated the potential of the ELM-based toxicity test for differentiating pathotypes among the highly diverse *C*. *cassiicola* species.

Several studies have described the genetic diversity of the *C*. *cassiicola* species but the biological features associated with the various genetic races often remain unknown. The electrolyte leakage test may thus be a useful method to ascribe physiological traits to the genetic groups among the *C*. *cassiicola* species, even though it only concerns the early stages of infection, *i*.*e*. the toxicity of the exudates. We have previously proposed a classification of *C*. *cassiicola* isolates based on phylogenetic clusters and toxin classes defined from the cassiicolin isoform(s) detected by PCR (Cas1 to Cas6), with the toxin class Cas0 corresponding to isolates without cassiicolin gene [10]. In the present study, the tested culture filtrates were of various origins (seven Asian and three African) and representative of four different isolate types: A4/Cas0, B4/Cas5, C/Cas1 and F/Cas0. The clustering we obtained based on the matrix of EL% data (Fig 4) identified two clusters within the treatments (Tr1 and Tr2), corresponding to weakly aggressive treatments (including the blanks) and aggressive treatments respectively, but these did not clearly match the genetic types. Filtrates in cluster Tr1 are of mixed types (F/Cas0, A4/Cas0 and B4/Cas5). Filtrates in cluster Tr2 are of two types only (C/Cas1 and A4/Cas0). Among the aggressive treatments, CCP (type C/Cas1) was found to be associated with the related Tox5 and Tox10 treatments (*i*.*e*. the purified toxin Cas1 extracted from CCP, at medium and high concentrations). By contrast, the toxin at low concentration (Tox1) was found to be associated with the weakly aggressive Tr1 filtrates, of unrelated type, which were probably more influenced by the initial scraping of the leaflets than by the fungal exudates. An ANOVA analysis performed on the reduced dataset corresponding to Tox1, Tox5, Tox10 and CCP responses alone (S3 Table) evidenced no clone x treatment interaction, indicating similar sensitivity profiles in response to either the purified toxin or the crude CCP filtrate. These results all together evidence that Cas1 is an important effector of CCP filtrate toxicity. Within cluster Tr2, CCP was clearly separated from the other filtrates, of type C/Cas1 (CCAM3) and A4/Cas0 (CSRI5, CCI6 and CCI13). Filtrate CCAM3 was less aggressive than CCP, mostly on the clones IRCA19, PB217 and PB254, although both filtrates were of the same type. It was previously shown that the cassiicolin gene (*Cas1*) was strictly identical in both isolates (no allelic variation) but under-expressed in CCAM3 compared to CCP, in good coherence with the lower aggressiveness of this isolate [9]. In our study, the CCAM3 filtrate, putatively poor in cassiicolin, behaved like the aggressive isolates of A4/Cas0, suggesting that they may share a common effector other than cassiicolin. C/Cas1 may be considered as one pathotype and A4/Cas0 as another, both allowing high aggressiveness but with different clonal sensitivity profiles. The Cas1 effector is probably a key element for differentiating these two pathotypes, based on both qualitative (presence/absence) and quantitative variations. However, the delineation of pathotypes in *C*. *cassiicola* remains unclear, since virulence in this species likely involves multiple effectors, either specific or shared by several isolates, with potential allelic variations and gene expression modulations.

### QTL associated with the response of rubber tree to *C*. *cassiicola* exudates

We present in this study a saturated genetic linkage map obtained from the rubber tree family PB260 x RRIM600 using SSR markers only. The total map length (2005 cM), average map length per chromosome (112 cM) and SSR marker order were in agreement with previously published rubber tree maps, on other families [19,22,48]. Parental maps have not been built in our work, because the colinearity between parental genomes has already been demonstrated [19]. The size of the full-sib family (191 genotypes) and the quality of the markers (2% distorted markers only) were sufficient to build a saturated map. Although large segments without mapped markers remain, this map was dense enough for efficient detection of QTL.

This is the first study describing QTL of sensitivity/tolerance to *C*. *cassiicola* exudates in *H*. *brasiliensis*. Six QTL located on five chromosomes were detected for seven isolates of various geographical origins and genetic types, with up to two QTL per treatment and moderate percentages of explanation of the total phenotypic variance (higher than 10% but lower than 20%), thus demonstrating the polygenic determinism of the EL% responses in the context of our study. This result is in agreement with recent studies in barley [49,50] which identified multiple QTL associated with the sensitivity to exudates (intercellular wash fluids) from the necrotrophic pathogen *Pyrenophora teres f*.*sp*. *teres*, in good correlation with susceptibility to the corresponding isolates. However, in contrast with our results, a recent study described a monogenic resistance to *C*. *cassiicola*-induced target leaf spot in cucumber [51]: a recessive gene (*cca-3*) associated with the resistance was fine-mapped and found to be co-localized with a CC-NB-ARC type resistance (R) gene. One single nucleotide polymorphism (SNP) leading to amino acid change in this candidate R gene differentiates the susceptible and the resistant lines.

Plants have developed a two-tiered immune system involving the recognition of pathogen-associated molecules by receptor proteins [52–56]. A first level of defense is the innate immune system which involves the perception of both pathogen-associated (non-self) molecular patterns (PAMPs) and danger-associated (self) molecular patterns (DAMPs), *via* surface-localized pattern recognition receptors (PRRs) [55,57]. Common fungal PAMPs are carbohydrates from the cell wall, including mannoproteins, phospholipomannan, beta-glucans and chitin, as well as enzymes such as xylanase or polygalacturonases. DAMPs are released from damaged plant tissues; they include extracellular ATP, peptides and cell wall or DNA fragments. The deployment of a set of defense mechanisms by the plant upon recognition of PAMPs or DAMPs by PRRs is known as PRR-triggered immunity (PTI). To bypass these broad spectrum defenses, pathogens deliver virulence effectors either to the plant cell apoplast, in order to block PAMP perception, or inside the cell where they interfere with PTI, resulting in susceptibility. Plants have evolved a second level of defense, triggered by the direct or indirect recognition of pathogen-produced effectors by intracellular and extracellular plant receptor R proteins. This specific “gene-for-gene” interaction usually triggers a strong and rapid hypersensitive response (HR) involving host-controlled programmed cell death, which in the case of biotrophic pathogens efficiently blocks the pathogen propagation. This model is known as effector-triggered immunity (ETI) [52]. However, in the case of necrotrophic pathogens adapted to colonize dying tissues, such programmed host cell death may favor, instead of limiting, pathogen development. The model involving the interaction between a necrotrophic effector (NE) and its cognate plant receptor is known as NE-triggered susceptibility (NETS) [49,58] as opposed to the ETI. Several genes conferring sensitivity to fungal necrotrophic effectors have been characterized [59–62]. All of them are single genes encoding NB-LRR receptor-like proteins. They confer dominant sensitivity to the necrotrophic effector and dominant susceptibility to the disease in their host plant.

The culture filtrates analyzed in our study are expected to be a complex mixture including both PAMPs (carbohydrates from the cell wall, hydrolytic enzymes) and effectors (secondary metabolites, small secreted proteins). It is therefore not surprising to observe multiple QTL, reflecting multiple interactions, when using the ELM-based toxicity test. However, even the cassiicolin toxin Cas1, purified to homogeneity from filtrate CCP, generated two QTL. Although the mechanism ruling Cas1 toxicity in rubber tree has not been elucidated yet, Cas1 shares many features with already characterized necrotrophic effectors: it is a small secreted cysteine-rich protein, with no known sequence homology in other species, generating necrosis with host- and cultivar-specialized profiles [6–9]. Several hypotheses may be raised to explain the polygenic determinism of sensitivity to Cas1 in rubber tree. One may be that two receptor-like proteins are required for the recognition of the Cas1 effector. The hemibiotrophic pathogen *Magnaporthe grisea*, infecting rice, provides examples of resistance conferred by pairs of receptor-like proteins [63]. Another hypothesis would be that Cas1 may interfere with PTI, presumably induced in that case by DAMPs as a result of leaf epidermis scrapping. It should be kept in mind that no QTL was revealed with the blank treatment (Tox0) although it similarly involved leaf scrapping. Therefore, it can be assumed that the two identified QTL are indeed associated with Cas1, potentially interfering with DAMP-triggered immunity, but not associated with wounding alone. Since PTI deploys a large set of defense mechanisms, Cas1 efficiency in interfering with PTI may vary among the progeny and reveal more than one QTL. Multiple QTL may reflect toxin interaction with various plant targets to disturb the normal functioning of the plant and/or detoxification processes aiming at limiting the toxin effects. As an example of toxin detoxification mechanism in maize, the Hm1 and/or Hm2 *loci* were found to encode a carbonyl reductase that detoxifies the HC-toxin, determinant of host-specificity and virulence of *Cochliobolus carbonum* [64]. Further characterization of the two cassiicolin-associated QTL regions would shed light on the plant mechanisms controlling sensitivity to this effector.

The fact that two QTL (g2-26 and g4-95) are common to both CCP filtrate and the purified cassiicolin Cas1 suggests that Cas1 is the major determinant of CCP filtrate toxicity. This was also evidenced by the response of 18 clones to CCP and three concentrations of toxin, which revealed similar sensitivity profiles whatever the treatment (Table 3 and S1 Fig). The Cas1 effector may be responsible for the high aggressiveness of isolate CCP, although we cannot exclude the existence of other effectors whose effects may be hidden by the effect of Cas1.

QTL profiles varied depending on the filtrates, suggesting the involvement of isolate-specialized toxicity factors, presumably effectors rather than PAMPs, since those are usually broadly conserved among pathogens of the same species. CCP clearly contrasts with the other filtrates as shown by the pairwise correlations between the treatments applied to the F1 progenies (Table 6) or by the clustering of the 15 treatments tested on 18 clones (Fig 4). And indeed, the two QTL associated with CCP (g2-26 and g4-95) were not shared by any other filtrate. All the other filtrates were positively correlated to each other (Table 6). QTL g4-32 may be interacting with a specific effector common to filtrates of type A4/Cas0 (CCI6, CCI13 and CSRI5). On the other hand, QTL g13-102 was detected with three isolates (CCAM3, CSB16 and CSRI5) of three different types (C/Cas1, B4/Cas5 and A4/Cas0 respectively) and may thus correspond to more general unspecific defense mechanisms. Alternatively, multiple sensitivity/insensitivity factors such as R proteins, each interacting with specific effectors from the different isolates, may be collocated at that position. This is a likely explanation knowing that resistance gene analogs (RGAs) are frequently clusterized in plant genomes [65–68].

Finally, two QTL identified in this study were localized in the same genomic region as QTL previously found associated with the tolerance to *Microcyclus ulei*, in other families. The QTL g2-26 associated with the response to filtrate CCP and Cas1 seems co-localized with the major *M2fx* QTL detected in the family PB260 x Fx2784 [21]. The QTL g13-102 detected for the CCAM3, CSB16 and CSRI5 culture filtrates seems co-localized with the major QTL *M13-1bn* detected in the family PB260 x RO38 [69]. However, considering the low precision of QTL localization, we do not know at this stage if common or distinct co-located factors are involved in the interaction between rubber tree and these two very different foliar pathogens (*M*. *ulei* being biotrophic and host-specific, while *C*. *cassiicola* is non biotrophic, with a large host range).

Globally, our results show that a limited number of relatively important genetic factors may be involved in the process of sensitivity/insensitivity of rubber clones to *C*. *cassiicola* exudates. The sensitivity alleles may come from the female parent (PB260) in the case of the main QTL identified in response to CCP or Cas1, from the male parent (RRIM600) in the case of the main QTL identified in response to TSB1, and from parental allelic interaction in the case of QTL identified in response to CCI6, CCI13, CSRI5, CSB16 and CCAM3 (Table 7).

## Conclusion

The indirect toxicity test based on induced electrolyte leakage measurements seems a practical and sensitive method for monitoring the early events of *H*. *brasiliensis* x *C*. *cassiicola* interaction, taking into account the genetic diversity of *C*. *cassiicola* without the risk of spreading new virulent strains in the environment.

Application of this method to the phenotyping of the PB260 x RRIM600 F1 family revealed the polygenic determinism of sensitivity/insensitivity to *C*. *cassiicola* exudates in this rubber tree family, and more specifically to the purified cassiicolin effector. It is proposed that the combined action of PAMPs contained in the filtrates and DAMPs resulting from leaf scrapping induces PTI which is then likely modulated by specific fungal molecules such as cassiicolin or yet unknown effectors.

The ELM-based toxicity test will be a useful tool for genetic investigations involving candidate effectors as well as for early selection. However, further studies are needed to evaluate the power of this test in predicting the susceptibility of rubber clones to the natural inoculum in field conditions.

## Supporting Information

S1 Fig Distribution of the EL% data for 18 clones depending on toxin concentration. The clones were treated with the purified toxin Cas1 at 1, 5 and 10 ng/μL (Tox1, Tox5, Tox10 respectively), the CCP filtrate from which Cas1 was extracted, as well as two blank treatments, water (Tox0) and the culture medium Cz. Top letters indicate the significance of differences between treatments (SNK test, risk α = 0.05).
(TIFF)

S1 Table Responses of eight rubber clones to filtrate application or conidial inoculation. Eight clones (GT1, PB217, PB260, RRIC100, RRIM600, IRCA18, IRCA41 and IRCA631) were treated with culture filtrates or spore suspensions from two isolates (CCP and CCI13), on detached leaves. Sensitivity to the culture filtrates was expressed as the induced electrolyte leakage EL%. Susceptibility in response to spore inoculation was expressed as the surface of leaf necrosis, in mm^2^. “se” are standard errors over three biological repeats. Superscript letters indicate the significance of differences between clones for each treatment (SNK test, risk α = 0.05).
(DOCX)

S2 Table Pearson correlation coefficients between treatments. Two isolates (CCI13 and CCP) were tested on eight clones following two test methods (filtrate application or conidial inoculation), on detached leaves, as described in Fig 3 and S1 Table. ‘*’ p<0.05; ‘**’ p<0.01.
(DOCX)

S3 Table ANOVA of the EL% response of 18 clones to the CCP culture filtrate and to the purified cassiicolin Cas1 at 1, 5 or 10 ng/μL (4 motifs). R^2^ = 0.70, ‘***’ p<0.001.
(DOCX)

## References

[1] Hora Júnior BTd, de Macedo DM, Barreto RW, Evans HC, Mattos CRR, Maffia LA, et al Erasing the Past: A New Identity for the Damoclean Pathogen Causing South American Leaf Blight of Rubber. PLoS ONE. 2014;9(8):e104750 10.1371/journal.pone.0104750 25126853 PMC4134235

[2] Deighton F. A preliminary list of fungi and diseases of plants from Sierra Leone. Kew Bulletin 1936:397–424.

[3] Ramakrishnan T, Pillay P. Leaf spot of rubber caused by *Corynespora cassiicola* (Berk. & Curt.) Wei. Rubber Board Bulletin. 1961;5:52–3.

[4] Newsam A. Plant Pathology Division Report Kuala Lumpur, Malaysia: Rubber Research Institute of Malaysia, 1961;63–70.

[5] Liyanage ADS, Jayasinghe CK, Liyanage NIS, Jayaratne AHR. *Corynespora* leaf spot disease of rubber (*Hevea brasiliensis*)—a new record. J Rubber Res Inst Sri Lanka. 1986;65:47–50.

[6] Breton F, Sanier C, d'Auzac J. Role of cassiicolin, a host-selective toxin, in pathogenicity of *Corynespora cassiicola*, causal agent of a leaf fall disease of Hevea. J Rubber Res. 2000;3(2):115–28.

[7] de Lamotte F, Duviau MP, Sanier C, Thai R, Poncet J, Bieysse D, et al Purification and characterization of cassiicolin, the toxin produced by *Corynespora cassiicola*, causal agent of the leaf fall disease of rubber tree. J Chromatogr B Analyt Technol Biomed Life Sci. 2007;849(1–2):357–62. 10.1016/j.jchromb.2006.10.051 17113837

[8] Barthe P, Pujade-Renaud V, Breton F, Gargani D, Thai R, Roumestand C, et al Structural analysis of cassiicolin, a host-selective protein toxin from *Corynespora cassiicola*. J Mol Biol. 2007;367(1):89–101. 10.1016/j.jmb.2006.11.086 17234212

[9] Déon M, Bourré Y, Gimenez S, Berger A, Bieysse D, de Lamotte F, et al Characterization of a cassiicolin-encoding gene from *Corynespora cassiicola*, pathogen of rubber tree (*Hevea brasiliensis*). Plant Science. 2012a;185-186(0):227–37. 22325885 10.1016/j.plantsci.2011.10.017

[10] Déon M, Fumanal B, Gimenez S, Bieysse D, Oliveira RR, Shuib SS, et al Diversity of the cassiicolin gene in *Corynespora cassiicola* and relation with the pathogenicity in *Hevea brasiliensis*. Fungal Biology. 2014;118(1):32–47. 10.1016/j.funbio.2013.10.011 24433675

[11] Silva WPK, Deverall BJ, Lyon BR. Molecular, physiological and pathological characterization of *Corynespora* leaf spot fungi from rubber plantations in Sri Lanka. Plant Pathol 1998;47(3):267–77.

[12] Atan S, Hamid NH. Differentiating races of *Corynespora cassiicola* using RAPD and internal transcribed spacer markers. J Rubber Res. 2003;(1):58–64.

[13] Silva WP, Karunanayake EH, Wijesundera RL, Priyanka UM. Genetic variation in *Corynespora cassiicola*: a possible relationship between host origin and virulence. Mycol Res. 2003;107:567–71. 12884953 10.1017/s0953756203007755

[14] Nghia NA, Kadir J, Sunderasan E, Puad Abdullah M, Malik A, Napis S. Morphological and inter simple sequence repeat (ISSR) markers analyses of *Corynespora cassiicola* isolates from rubber plantations in Malaysia. Mycopathologia. 2008;166(4):189–201. 10.1007/s11046-008-9138-8 18568417

[15] Dixon LJ, Schlub RL, Pernezny K, Datnoff LE. Host specialization and phylogenetic diversity of *Corynespora cassiicola*. Phytopathology. 2009;99(9):1015–27. 10.1094/PHYTO-99-9-1015 19671003

[16] Qi Y, Xie Y, Zhang X, Pu J, Zhang H, Huang S, et al Molecular and pathogenic variation identified among isolates of *Corynespora cassiicola*. Mol Biotechnol. 2009;41(2):145–51. 10.1007/s12033-008-9109-9 18841502

[17] Hieu ND, Nghia NA, Chi VTQ, Dung P. Genetic diversity and pathogenicity of *Corynespora cassiicola* isolates from rubber trees and other hosts in Vietnam. J Rubber Res. 2014;17(3):187–203.

[18] Shuib SS, Déon M, Mahyuddin MM, Izhar A, Fumanal B, Sunderasan E, et al Cassiicolin genes among *Corynespora* isolates from rubber plantations in Malaysia. J. Rubber Res. 2015;18(2):109–26.

[19] Lespinasse D, Rodier-Goud M, Grivet L, Leconte A, Legnate H, Seguin M. A saturated genetic linkage map of rubber tree (*Hevea spp*) based on RFLP, AFLP, microsatellite and isozyme markers. Theor Appl Genet. 2000a;100:127–38.

[20] Lespinasse D, Grivet L, Troispoux V, Rodier-Goud M, Pinard F, Seguin M. Identification of QTLs involved in the resistance to South American leaf blight (*Microcyclus ulei*) in the rubber tree. Theoretical and Applied Genetics. 2000b;100(6):975–84.

[21] Le Guen V, Guyot J, Mattos CRR, Seguin M, Garcia D. Long lasting rubber tree resistance to *Microcyclus ulei* characterized by reduced conidial emission and absence of teleomorph. Crop protection. 2008;27(12): 1498–503.

[22] Le Guen V, Garcia D, Doaré F, Mattos CR, Condina V, Couturier C, et al A rubber tree’s durable resistance to *Microcyclus ulei* is conferred by a qualitative gene and a major quantitative resistance factor. Tree Genetics & Genomes. 2011;7(5):877–89.

[23] Le Guen V, Garcia D, Mattos C, Fouet O, Doaré F, Condina V, et al A newly identified locus controls complete resistance to *Microcyclus ulei* in the Fx2784 rubber clone. Tree Genetics & Genomes. 2013;9(3):805–12.

[24] Jayasinghe CK. Corynespora leaf fall: the most challenging rubber disease in Asian and African continents. 2000;42:56–64.

[25] Manju MJ, Vinod KK, Idicula SP, Kuruvilla J, Nazeer MA, Benagi VI. Susceptibility of *Hevea brasiliensis* clones to *Corynespora* Leaf Fall disease. J Mycol Pl Pathol. 2010;40(4):603–9.

[26] Fernando THPS, Jayasinghe CK, Wijesundera RLC, Silva WPK, Nishantha EADN. Evaluation of screening methods against Corynespora leaf fall disease of rubber tree (*Hevea brasiliensis*). Journal of Plant Diseases and Protection. 2010;117(1):24–9.

[27] White T, Bruns T, Lee S, Taylor J. Amplification and direct sequencing of fungal ribosomal RNA genes for phylogenetics In: Innis M, Gelfand D, Shinsky J, White T, editors. PCR Protocols: A Guide to Methods and Applications. San Diego: Academic Press; 1990 p. 315–22.

[28] Lutts S, Kinet JM, Bouharmont J. NaCl-induced senescence in leaves of rice (*Oryza sativa L*.) cultivars differing in salinity resistance. Annals of Botany. 1996;78(3):389–98.

[29] Herbette S, Menn AL, Rousselle P, Ameglio T, Faltin Z, Branlard G, et al Modification of photosynthetic regulation in tomato overexpressing glutathione peroxidase. Biochim Biophys Acta. 2005;1724(1–2):108–18. 10.1016/j.bbagen.2005.04.018 15921856

[30] Mai J, Herbette S, Vandame M, Kositsup B, Kasemsap P, Cavaloc E, et al Effect of chilling on photosynthesis and antioxidant enzymes in Hevea brasiliensis Muell. Arg. Trees—Structure and Function. 2009;23(4):863–74.

[31] Hallé F, Martin R. Etude de la croissance rythmique chez l'hévéa (*Hevea brasiliensis*) Müll. Arg., (Euphorbiacées, crotonoïdées). Adansonia. 1968;8:475–503.

[32] Chee KH. Studies on sporulation, pathogenicity and epidemiology of *Corynespora cassiicola* on Hevea rubber. 1988;3(1):21–9.

[33] Seguin M, Rodier-Goud M, Lespinasse D. Mapping SSR markers in rubber tree (Hevea brasiliensis) facilitated and enhanced by heteroduplex formation and template mixing. Plant and Animal Genomes V Conference; San Diego, USA1997. p. 66.

[34] Le Guen V, Gay C, Xiong TC, Souza LM, Rodier-Goud M, Seguin M. Development and characterization of 296 new polymorphic microsatellite markers for rubber tree (*Hevea brasiliensis*). Plant Breeding. 2011;130(2):294–6.

[35] Cubry P, Pujade-Renaud V, Garcia D, Espeout S, Guen Vl, Granet F, et al Development and characterization of a new set of 164 polymorphic EST-SSR markers for diversity and breeding studies in rubber tree (*Hevea brasiliensis* Mull. Arg.). Plant Breeding. 2014;133(3):419–26.

[36] Risterucci AM, Grivet L, N'Goran JAK, Pieretti I, Flament M-H, Lanaud C. A high-density linkage map of *Theobroma cacao* L. Theor Appl Genet. 2000;101:948–55. 10.1007/BF00223910 24169987

[37] Van Ooijen JW. JoinMap 4, Software for the mapping of quantitative trait loci in experimental populations. Wageningen, Netherlands: Kyazma, B V; 2006.

[38] Stam P. Construction of integrated genetic linkage maps by means of a new computer package: Join Map. The Plant Journal. 1993;3(5):739–44.

[39] Van Ooijen JW. MapQTL 6, Software for the mapping of quantitative trait loci in experimental populations of diploid species Wageningen, Netherlands: Kyazma, B V; 2009.

[40] Churchill GA, Doerge RW. Empirical threshold values for quantitative trait mapping. Genetics. 1994;138(3):963–71. 7851788 10.1093/genetics/138.3.963 PMC1206241

[41] Morton NE. Sequential tests for the detection of linkage. American journal of human genetics. 1955;7(3):277–318. 13258560 PMC1716611

[42] Lander ES, Botstein D. Mapping mendelian factors underlying quantitative traits using RFLP linkage maps. Genetics. 1989;121(1):185–99. 2563713 10.1093/genetics/121.1.185 PMC1203601

[43] Jansen RC. Interval mapping of multiple quantitative trait loci. Genetics. 1993;135(1):205–11. 8224820 10.1093/genetics/135.1.205 PMC1205619

[44] Jansen RC, Stam P. High resolution of quantitative traits into multiple loci via interval mapping. Genetics. 1994;136(4):1447–55. 8013917 10.1093/genetics/136.4.1447 PMC1205923

[45] Déon M, Scomparin A, Tixier A, Mattos C, Leroy T, Seguin M, et al First characterization of endophytic *Corynespora cassiicola* isolates with variant cassiicolin genes recovered from rubber trees in Brazil. Fungal Diversity. 2012b;54(1):87–99.

[46] Ritter E, Gebhardt C, Salamini F. Estimation of recombination frequencies and construction of RFLP linkage maps in plants from crosses between heterozygous parents. Genetics. 1990;125(3):645–54. 1974227 10.1093/genetics/125.3.645 PMC1204090

[47] Jayasinghe CK. Corynespora leaf fall and future of the leading rubber clones in the world. 30 ref. 2002:5–11.

[48] Rattanawong R, Teerawatanasuk K, Prapan K, Kasemsap P, Clément-Demange A, Lekawipat N, editors. Application of QTL mapping for early selection on growth and latex yield traits in rubber breeding. IRRDB International Rubber Conference; 2011 14–17 December, Chiang Mai, Thailand.

[49] Liu Z, Holmes DJ, Faris JD, Chao S, Brueggeman RS, Edwards MC, et al Necrotrophic effector-triggered susceptibility (NETS) underlies the barley-*Pyrenophora teres f*. *teres* interaction specific to chromosome 6H. Mol Plant Pathol. 2015;16(2):188–200. 10.1111/mpp.12172 25040207 PMC6638325

[50] Gao Y, Faris JD, Liu Z, Kim YM, Syme RA, Oliver RP, et al Identification and Characterization of the SnTox6-Snn6 Interaction in the *Parastagonospora nodorum*-Wheat Pathosystem. Mol Plant Microbe Interact. 2015;28(5):615–25. 10.1094/MPMI-12-14-0396-R 25608181

[51] Wen C, Mao A, Dong C, Liu H, Yu S, Guo Y-D, et al Fine genetic mapping of target leaf spot resistance gene cca-3 in cucumber, *Cucumis sativus* L. Theoretical and Applied Genetics. 2015;128(12):2495–506. 10.1007/s00122-015-2604-z 26385372

[52] Jones JD, Dangl JL. The plant immune system. Nature. 2006;444(7117):323–9. 10.1038/nature05286 17108957

[53] De Wit PJGM, Mehrabi R, Van Den Burg HA, Stergiopoulos I. Fungal effector proteins: past, present and future. Mol Plant Pathol. 2009;10(6):735–47. 10.1111/j.1364-3703.2009.00591.x 19849781 PMC6640362

[54] Dangl JL, Horvath DM, Staskawicz BJ. Pivoting the plant immune system from dissection to deployment. Science. 2013;341(6147):746–51. 10.1126/science.1236011 23950531 PMC3869199

[55] Zipfel C. Plant pattern-recognition receptors. Trends in immunology. 2014;35(7):345–51. 10.1016/j.it.2014.05.004 24946686

[56] Keller H, Boyer L, Abad P. Disease susceptibility in the Zig-Zag model of host-microbe interactions: only a consequence of immune suppression? Mol Plant Pathol. 2016;17(4):475–9. 10.1111/mpp.12371 26788791 PMC6638450

[57] Heil M, Land WG. Danger signals—damaged-self recognition across the tree of life. Front Plant Sci. 2014;5:578 10.3389/fpls.2014.00578 25400647 PMC4215617

[58] Friesen TL, Meinhardt SW, Faris JD. The *Stagonospora nodorum*-wheat pathosystem involves multiple proteinaceous host-selective toxins and corresponding host sensitivity genes that interact in an inverse gene-for-gene manner. Plant J. 2007;51(4):681–92. 10.1111/j.1365-313X.2007.03166.x 17573802

[59] Lorang JM, Sweat TA, Wolpert TJ. Plant disease susceptibility conferred by a "resistance" gene. Proc Natl Acad Sci U S A. 2007;104(37):14861–6. 10.1073/pnas.0702572104 17804803 PMC1976202

[60] Sweat TA, Wolpert TJ. Thioredoxin h5 is required for victorin sensitivity mediated by a CC-NBS-LRR gene in Arabidopsis. Plant Cell. 2007;19(2):673–87. 10.1105/tpc.106.047563 17322408 PMC1867327

[61] Nagy ED, Bennetzen JL. Pathogen corruption and site-directed recombination at a plant disease resistance gene cluster. Genome Res. 2008;18(12):1918–23. 10.1101/gr.078766.108 18719093 PMC2593579

[62] Faris JD, Zhang Z, Lu H, Lu S, Reddy L, Cloutier S, et al A unique wheat disease resistance-like gene governs effector-triggered susceptibility to necrotrophic pathogens. Proc Natl Acad Sci U S A. 2010;107(30):13544–9. 10.1073/pnas.1004090107 20624958 PMC2922177

[63] Cesari S, Thilliez G, Ribot C, Chalvon V, Michel C, Jauneau A, et al The rice resistance protein pair RGA4/RGA5 recognizes the *Magnaporthe oryzae* effectors AVR-Pia and AVR1-CO39 by direct binding. Plant Cell. 2013;25(4):1463–81. 10.1105/tpc.112.107201 23548743 PMC3663280

[64] Walton JD. HC-toxin. Phytochemistry. 2006;67(14):1406–13. 10.1016/j.phytochem.2006.05.033 16839576

[65] Kim J, Lim CJ, Lee BW, Choi JP, Oh SK, Ahmad R, et al A genome-wide comparison of NB-LRR type of resistance gene analogs (RGA) in the plant kingdom. Molecules and cells. 2012;33(4):385–92. 10.1007/s10059-012-0003-8 22453776 PMC3887800

[66] Yang L, Li D, Li Y, Gu X, Huang S, Garcia-Mas J, et al A 1,681-locus consensus genetic map of cultivated cucumber including 67 NB-LRR resistance gene homolog and ten gene loci. BMC Plant Biol. 2013;13:53 10.1186/1471-2229-13-53 23531125 PMC3626583

[67] Perazzolli M, Malacarne G, Baldo A, Righetti L, Bailey A, Fontana P, et al Characterization of resistance gene analogues (RGAs) in apple (*Malus × domestica Borkh*.) and their evolutionary history of the rosaceae family. PLoS ONE. 2014;9(2):e83844 10.1371/journal.pone.0083844 24505246 PMC3914791

[68] Chen JY, Huang JQ, Li NY, Ma XF, Wang JL, Liu C, et al Genome-wide analysis of the gene families of resistance gene analogues in cotton and their response to Verticillium wilt. BMC Plant Biol. 2015;15:148 10.1186/s12870-015-0508-3 26084488 PMC4471920

[69] Le Guen V, Lespinasse D, Oliver G, Rodier-Goud M, Pinard F, Seguin M. Molecular mapping of genes conferring field resistance to South American Leaf Blight (*Microcyclus ulei*) in rubber tree. Theor Appl Genet. 2003;108(1):160–7. 10.1007/s00122-003-1407-9 14504743

