# Supplementary figures and images for: Genetic Determinism of Sensitivity to Corynespora cassiicola Exudates in Rubber Tree (Hevea brasiliensis)
Source: PLoS One. 2016 Oct 13;11(10):e0162807. doi: 10.1371/journal.pone.0162807 (PMC5063417; doi:10.1371/journal.pone.0162807)

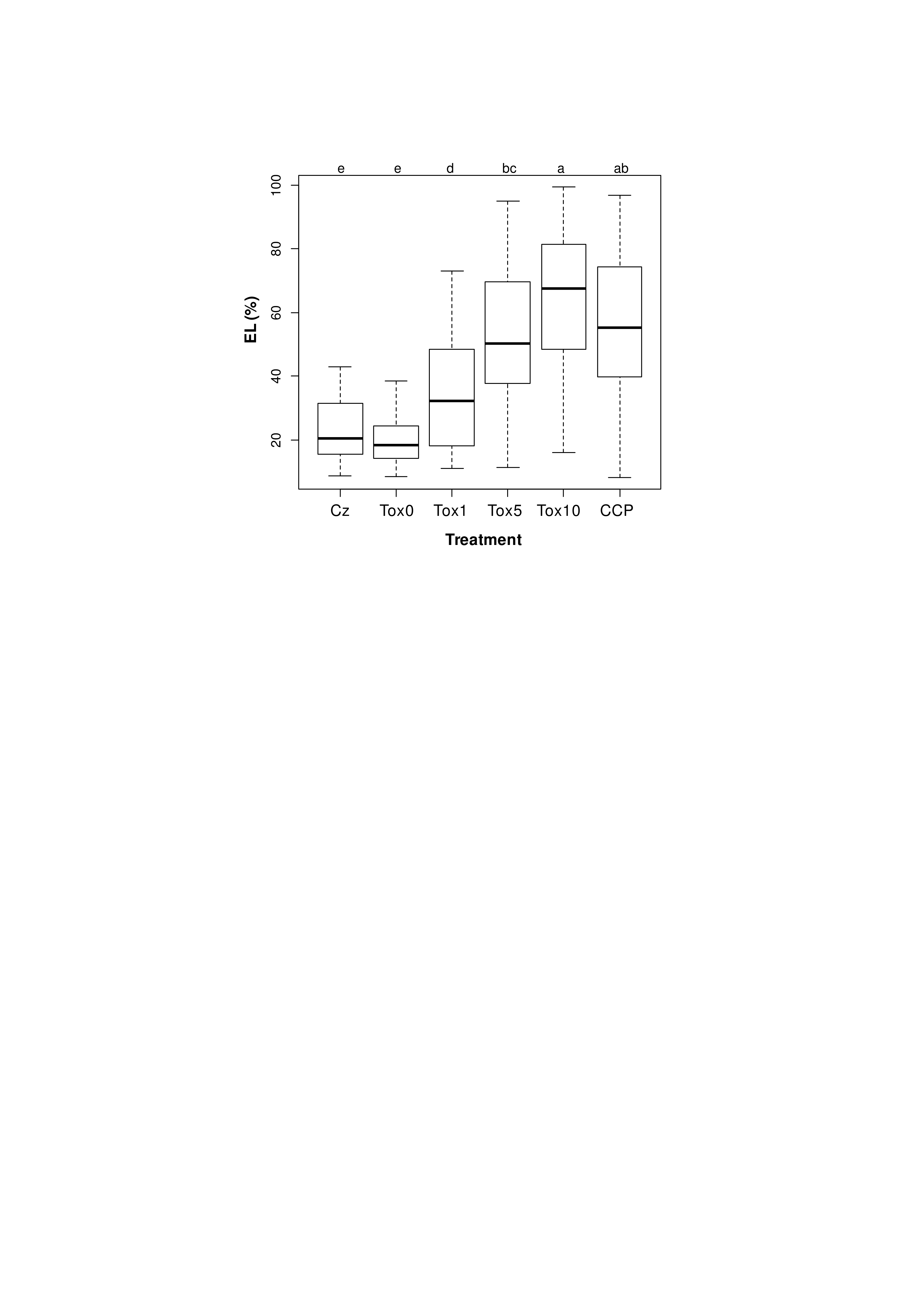

Supplement: S1 Fig — The clones were treated with the purified toxin Cas1 at 1, 5 and 10 ng/μL (Tox1, Tox5, Tox10 respectively), the CCP filtrate from which Cas1 was extracted, as well as two blank treatments, water (Tox0) and the culture medium Cz. Top letters indicate the significance of differences between treatments (SNK test, risk α = 0.05). (TIFF) [file pone.0162807.s001.tiff]
