# Supplementary material for: Genetic Determinism of Sensitivity to Corynespora cassiicola Exudates in Rubber Tree (Hevea brasiliensis)
Source: PLoS One. 2016 Oct 13;11(10):e0162807. doi: 10.1371/journal.pone.0162807 (PMC5063417; doi:10.1371/journal.pone.0162807)
Supplement: S1 Table — Eight clones (GT1, PB217, PB260, RRIC100, RRIM600, IRCA18, IRCA41 and IRCA631) were treated with culture filtrates or spore suspensions from two isolates (CCP and CCI13), on detached leaves. Sensitivity to the culture filtrates was expressed as the induced electrolyte leakage EL%. Susceptibility in response to spore inoculation was expressed as the surface of leaf necrosis, in mm2. “se” are standard errors over three biological repeats. Superscript letters indicate the significance of differences between clones for each treatment (SNK test, risk α = 0.05). (DOCX) [file pone.0162807.s002.docx]

**S1 Table. Responses of eight rubber clones to filtrate application or conidial inoculation.**

| Treatment | CCP isolate | | | | CCI13 isolate | | | |
| --- | --- | --- | --- | --- | --- | --- | --- | --- |
|  | Filtrate application | | Conidia inoculation | | Filtrate application | | Conidia inoculation | |
| Clone | Mean | se | Mean | se | Mean | se | Mean | se |
| IRCA631 | 76.4^a^ | 7.5 | 27.5^a^ | 1.4 | 40.7^a^ | 9.3 | 17.9^a^ | 1.5 |
| PB260 | 59.7^b^ | 10.4 | 21.7^b^ | 1.4 | 37.4^b^ | 10.3 | 10.6^bc^ | 1.4 |
| IRCA18 | 54.4^b^ | 3.8 | 21.6^b^ | 1.8 | 28.4^b^ | 10.3 | 8.4^c^ | 1.7 |
| PB217 | 42.0^c^ | 8.1 | 19.6^b^ | 2.1 | 10.8^c^ | 4.0 | 6.9^c^ | 1.6 |
| RRIC100 | 37.5^cd^ | 9.6 | 11.7^c^ | 1.4 | 33.9^b^ | 8.2 | 13.6^b^ | 1.8 |
| RRIM600 | 20.4^de^ | 13.5 | 8.8^c^ | 1.4 | 32.8^b^ | 9.9 | 8.9^c^ | 1.5 |
| GT1 | 15.3^e^ | 1.5 | 1.0^d^ | 1.8 | 14.4^c^ | 2.4 | 1.4^d^ | 1.7 |
| IRCA41 | 11.3^e^ | 0.6 | 1.3^d^ | 2.0 | 15.3^c^ | 3.9 | 0.0^d^ | 2.0 |

Eight clones (GT1, PB217, PB260, RRIC100, RRIM600, IRCA18, IRCA41 and IRCA631) were treated with culture filtrates or spore suspensions from two isolates (CCP and CCI13), on detached leaves. Sensitivity to the culture filtrates was expressed as the induced electrolyte leakage EL%. Susceptibility in response to spore inoculation was expressed as the surface of leaf necrosis, in mm^2^. “se” are standard errors over three biological repeats. Superscript letters indicate the significance of differences between clones for each treatment (SNK test, risk α= 0.05).
