# Supplementary material for: Genetic Determinism of Sensitivity to Corynespora cassiicola Exudates in Rubber Tree (Hevea brasiliensis)
Source: PLoS One. 2016 Oct 13;11(10):e0162807. doi: 10.1371/journal.pone.0162807 (PMC5063417; doi:10.1371/journal.pone.0162807)
Supplement: S2 Table — Two isolates (CCI13 and CCP) were tested on eight clones following two test methods (filtrate application or conidial inoculation), on detached leaves, as described in Fig 3 and S1 Table. ‘*’ p<0.05; ‘**’ p<0.01. (DOCX) [file pone.0162807.s003.docx]

**S2 Table. Pearson correlation coefficients between treatments**.

| Variables |  | Filtrate application | | Conidial inoculation | |
| --- | --- | --- | --- | --- | --- |
|  | Isolates | CCI13 | CCP | CCI13 | CCP |
| Filtrate application | CCI13 | 1 |  |  |  |
|  | CCP | 0.64 | 1 |  |  |
| Conidia inoculation | CCI13 | 0.84* | 0.81 | 1 |  |
|  | CCP | 0.56 | 0.97** | 0.78 | 1 |

Two isolates (CCI13 and CCP) were tested on eight clones following two test methods (filtrate application or conidial inoculation), on detached leaves, as described in Fig 3 and S1 Table. ‘*’ p<0.05; ‘**’ p<0.01.
