# Supplementary material for: Genetic Determinism of Sensitivity to Corynespora cassiicola Exudates in Rubber Tree (Hevea brasiliensis)
Source: PLoS One. 2016 Oct 13;11(10):e0162807. doi: 10.1371/journal.pone.0162807 (PMC5063417; doi:10.1371/journal.pone.0162807)
Supplement: S3 Table — R2 = 0.70, ‘***’ p<0.001. (DOCX) [file pone.0162807.s004.docx]

**S3 Table. ANOVA of the EL% response of 18 clones to the CCP culture filtrate and to the purified cassiicolin Cas1 at 1, 5 or 10 ng/µL (4 motifs).**

| Component | Df | Sum Sq | Mean Sq | F value | Pr (>F) |
| --- | --- | --- | --- | --- | --- |
| block | 2 | 301 | 150.7 | 0.7 | 0.5 |
| clone | 17 | 76312 | 4488.9 | 20.2 | < 2.2e-16*** |
| motif | 3 | 24844 | 8281.2 | 37.3 | < 2.2e-16*** |
| clone:motif | 51 | 16096 | 315.6 | 1.4 | 0.05 |
| Residuals | 218 | 48420 | 222.1 |  |  |

R^2^ = 0.70, ‘***’ p<0.001.
